# Hyaluronic acid−CD44 signaling from decidual stromal cells orchestrates dNK1 differentiation and immune tolerance in early pregnancy

**DOI:** 10.3389/fimmu.2026.1777567

**Published:** 2026-03-25

**Authors:** Di Wang, Jiawei Zhu, Qingzhi Wang, Lingyun Shi, Yehua He, Zili Jin, Wenjun Chen, Xiaocong Chen, Shenjun Chen, Hong Li, Ce Zhang, Rui Zhu

**Affiliations:** 1 Center for Human Reproduction and Genetics, The Affiliated Suzhou Hospital of Nanjing Medical University, Suzhou Municipal Hospital, Gusu School, Nanjing Medical University, Suzhou, China; 2 Medical Science and Technology Innovation Center, Central Laboratory, The Affiliated Suzhou Hospital of Nanjing Medical University, Suzhou Municipal Hospital, Gusu School, Nanjing Medical University, Suzhou, China; 3 Reproductive Medicine Center, The Fourth Affiliated Hospital of Soochow University (Suzhou DUSHU LAKE HOSPITAL), Suzhou, China

**Keywords:** CD44, decidual natural killer (dNK) cells, decidual stromal cells, hyaluronan synthase 2, hyaluronic acid

## Abstract

**Introduction:**

Spontaneous abortion (SA) is closely associated with immune homeostasis of decidual natural killer (dNK) cells at the maternal-fetal interface, yet how decidual stromal cells (DSCs) educate NK cells remains incompletely understood. Here, we showed that DSC-derived hyaluronic acid (HA) shapes NK cell residency and cytotoxicity in early pregnancy.

**Methods:**

Reanalysis of single-cell RNA sequencing data and functional assays identified DSCs as the major source of HAS2-dependent HA. We assessed dNK phenotypes and functions in SA versus normal pregnancies, and tested HA -CD44 effects using high-molecular-weight HA (HMW-HA) stimulation, HAS2 knockdown in DSCs, and CD44 blockade, with partial rescue by exogenous HMW-HA. Canonical Wnt activation and *FOSL2* upregulation were further evaluated by western blotting to examine downstream signaling.

**Results:**

Our findings revealed a reduced proportion of dNK cells, impaired adhesion molecule expression, and increased cytotoxicity in SA compared with normal pregnancies. HMW-HA from DSCs, via engagement of CD44 on NK cells, promoted the phenotypic transition of peripheral NK cells into CD49a^+^ tissue-resident dNK-like cells, increased adhesion, and shifted cytokine production toward an immune-tolerant profile. HAS2 knockdown in DSCs or CD44 blockade reduced CD49a expression, expanded highly cytotoxic CD44^high^ subset, and disrupted the balance among dNK1/dNK2/dNK3-like subsets, effects that were partially rescued by exogenous HMW-HA. Mechanistically, HA/CD44 signaling activated canonical Wnt pathways and upregulated the dNK1-associated transcription factor *FOSL2*, driving differentiation toward a low-cytotoxic dNK1-like phenotype.

**Discussion:**

These findings define a DSC-centered HA/HAS2 -CD44 -Wnt -*FOSL2* axis that remodels NK cells and supports maternal-fetal immune tolerance, providing potential targets for preventing SA and related pregnancy complications.

## Introduction

Spontaneous abortion (SA), defined as fetal loss before 28 weeks of gestation, is a prevalent complication in early pregnancy, affecting approximately 10% to 15% of clinically recognized pregnancies globally (1). The successful establishment and maintenance of a normal pregnancy are intricately linked to the homeostasis of the decidual immune microenvironment. Decidual natural killer (dNK) cells, constituting 50%-70% of the decidual immune cells (DICs) population at the maternal-fetal interface, are crucial for sustaining a normal pregnancy (2). Their multifaceted roles encompass promoting placental formation (3, 4), maintaining maternal-fetal immune tolerance (5–8), facilitating embryonic development (9, 10), providing anti-infection protection (11, 12), and maintaining pregnancy memory (13, 14). Characterized by low cytotoxicity and high secretory activity, dNK cells are predominantly CD56^bright^CD16^-^ (15–17). Recent single-cell sequencing (scRNA-seq) studies have further categorized them into three subtypes: dNK1, dNK2, and dNK3, with dNK1 being most actively involved in communication with extravillous trophoblasts (EVTs) (18). It has been observed that dNK cells not only expand *in situ* from uterine NK (uNK) cells post-conception but also migrate from a subset of peripheral NK (pNK) cells to the maternal-fetal interface, where they undergo transformation into dNK cells (19–23). However, most existing research has primarily focused on the involvement of trophoblasts in this pNK to dNK transition, while the contribution of decidual stromal cells (DSCs) has been less explored (24–28).

At the maternal-fetal interface, DSCs are the primary source of hyaluronic acid (HA), a glycosaminoglycan and a major component of the extracellular matrix, with its synthesis predominantly mediated by hyaluronan synthase 2 (HAS2) (29). Our previous studies have demonstrated that HA can instruct the polarization of decidual macrophages towards the M2 phenotype (30). Given the critical and more abundant presence of NK cells at this vital interface, it is plausible that DSC-derived HA also exerts a modulatory effect on these cells; however, its precise role in the remodeling of NK cells and the induction of their tolerogenic phenotype remains largely unexplored. Therefore, this study employed flow cytometry, RT-qPCR, and immunofluorescence to investigate the transformation of pNK cells into dNK-like cells under the instruction of DSCs. We further constructed a DSC model with HAS2 knockdown (HAS2KD) and blocked the CD44 receptor on the surface of NK cells to elucidate the role of the HA/CD44 axis. In parallel, we reanalyzed previously published scRNA-seq datasets to characterize the transcriptional landscape and developmental trajectory of NK cells and to explore the mechanisms by which HA instructs NK cell differentiation. This research aims to delineate the effects and underlying mechanisms of DSCs on NK cells at the maternal-fetal interface, thereby offering novel insights for the therapeutic strategies of SA or other pregnancy-related diseases stemming from maternal-fetal immune disorders.

## Materials and methods

All human samples were collected from the Affiliated Suzhou Hospital of Nanjing Medical University, with informed consent obtained from all participants. The study enrolled naturally conceived pregnant women, aged 18–39 years, with a gestational age ranging from 6 to 10 weeks. Eligibility criteria included no pregnancy-related comorbidities, no adverse history of pregnancy, and no reported medication during pregnancy. The natural pregnancy group (*n*=36) comprised women undergoing pregnancy termination for personal or social reasons. Conversely, the SA group (*n*=36) included patients diagnosed with unexplained clinical miscarriages, following the rigorous exclusion of genetic, anatomical and endocrine abnormalities, autoimmune diseases, infections, poor health habits.

### Isolation of human NK cells

Peripheral blood samples were collected from the normal pregnancy group via percutaneous venipuncture. Peripheral blood mononuclear cells (PBMCs) were subsequently isolated using Ficoll density gradient centrifugation (TBD, LTS1077-1), with PBMCs recovered from the interface layer. CD56^+^ NK cells were then purified from these PBMCs using a negative immuno-magnetic NK cell isolation kit (CD56^+^CD3^-^) (Miltenyi Biotech, 130-092-661).

Human decidual tissues were obtained from patients undergoing surgical termination of pregnancy for psychosocial reasons or from SA patients. After eliminating vascular tissues, fresh deciduas were thoroughly minced and enzymatically digested with a mixture of collagenase IV (2 mg/mL) (biosharp, BS165), dispase II (1 mg/mL) (biosharp, BS310), and DNase I (30 U/mL) (biosharp, BS137). The resulting digested tissues were passed through 100- and 300-mesh sieves to yield a single-cell suspension of total decidual cells. These cells underwent density gradient centrifugation using a Percoll gradient (20%, 40%, and 60%) (biosharp, BS909) to obtain DICs, which were enriched at the interface between the 40% and 60% layers. Following a 4-hour culture on plastic plates, non-adherent cells were collected. dNK cells were further enriched from these non-adherent cells using the same negative immuno-magnetic NK cell isolation kit (CD56^+^CD3^-^). Flow cytometry analysis confirmed that the purity of both isolated dNK and pNK cells exceeded 95% (**Supplementary Figure 1B**).

### Cell culture

Endometrial stromal cells (ESCs), a generous gift from Professor Mingqing Li (Shanghai Jiao Tong University), were maintained in phenol red-free F12K Ham medium (Sigma, D2906) supplemented with 1.5 mg/mL sodium bicarbonate (Aladdin, S118660), 1% insulin-transferrin-selenium-solution (Gibco, 41400045), and 10% FBS (Vivacell, C3830-0500). The NK92MI cell line (Boster, CX0331), a peripheral NK (pNK) cell surrogate expressing CD56^+^CD3^-^, was cultured in α-Dulbecco’s Modified Eagle’s Medium (α-MEM) (KeyGen, KGL1605-500) supplemented with 12.5% FBS (Vazyme, F102-03), 12.5% HS (Solarbio, S9050), 0.2 mM inositol (Solarbio, I8050), 0.1 mM β-mercaptoethanol (Macklin, M6230), and 0.02 mM folic acid (Solarbio, IF0180). The JEG-3 choriocarcinoma cell line (Boster, CX0091), serving as an extravillous trophoblast (EVT) model due to its similar human leukocyte antigen repertoire, was cultured in Modified Eagle’s Medium (MEM) containing 10% FBS. Additionally, the K562 cell line (Boster, CX0091), a standard target for NK cell cytotoxicity assays, was cultured in Roswell Park Memorial Institute (RPMI)-1640 (KeyGen, KGL1502-500) supplemented with 10% FBS (Vazyme, F101-03).

All cell cultures were maintained in a humidified incubator at 37 °C with 5% CO_2_.

### Cell treatment

To induce decidualization, ESCs were treated with 0.2 mM 8-bromo-cAMP (MedChemExpress, HY-2306), 1 μM medroxyprogesterone acetate (MedChemExpress, HY-B0469), and 10 nM estradiol (MedChemExpress, HY-B0141) for 48 hours. Successful decidualization was confirmed by the significant up-regulated expression of prolactin (**Supplementary Figure 2A**).

This study established three types of direct co-culture systems, all maintaining a 1:1 ratio of non-immune to immune cells. The primary system involved the co-culture of DSCs and NK92MI cells. Specifically, 1 × 10^5^ NK92MI cells were added to 1 × 10^5^ DSCs that had been pre-cultured for 24 hours, with the medium supplemented with folic acid and inositol. To dissect the molecular mechanisms, cells were treated with HMW-HA (100 μg/mL; MedChemExpress, HY-B0633A) or peptides (HA inhibitory vs. control, 100 μg/mL; Bioss; sequences in **Supplementary Table 1**) to evaluate the role of HA in NK cell education. To determine the signaling pathways involved, we used an anti-CD44 blocking antibody (30 μg/mL; BioxCell, BE0262) to block CD44, and IWP-2 (50 μM; MedChemExpress, HY-13912) to inhibit the canonical Wnt pathway.

In addition to the DSC-NK system, two other direct co-culture configurations were employed. First, a JEG-3 and NK92MI co-culture was established using the same methodology (1 × 10^5^ JEG-3 cells co-cultured with 1 × 10^5^ NK92MI cells). Second, a triple co-culture system (DSC + JEG-3 + NK92MI) was developed. For this, 1 × 10^5^ JEG-3 cells were added to 1 × 10^5^ pre-adherent DSCs for 6 hours; subsequently, the medium was replaced, and 2 × 10^5^ NK92MI cells were added for an additional 24 hours of co-culture.

### Cell supernatant treatment

Cell supernatants with specific molecular weight fractions and heat-inactivated proteins were prepared as follows. After 24 hours of culture in serum-free medium, the supernatant was collected and subjected to ultrafiltration using an ultrafiltration tube (Millipore, UFC910096) to obtain fractions with molecular weights of ≥ 300 kD and < 300 kD. For heat-inactivation, the cell supernatant was heated at 56 °C for 30 minutes. All prepared cell supernatants were subsequently adjusted to a final protein concentration of 100 μg/mL, as determined by a BCA protein assay kit (Vazyme, E112-02).

### Cell transfection

HAS2 KD and control plasmids (negative control, NC) were procured from Wuyuanbio. Cells cultured in 6-well plates to 80% confluence were then transfected with the respective plasmids using EZ Transfection Reagent (Life-iLab, AC04L091). Following a 6-hour incubation at 37 °C, cells were transferred to either fresh medium or conditioned medium for an additional 24 hours of culture. The transfection efficiency was assessed 48 hours later via GFP fluorescence intensity and Western blotting (WB).

### Flow cytometry assays

Human monoclonal antibodies (**Supplementary Table 2**) were utilized for measuring cell surface markers. For intracellular staining of cytokines and Ki67, cells were initially subjected to surface staining, then permeabilized using the Permeabilization Buffer from the Foxp3 Transcription Factor Staining Buffer Set (Thermo, 00-5523-00) according to the manufacturer’s instructions. Flow cytometry was performed on BD FACS Celesta instruments (BD Biosciences) as per the manufacturer’s instructions. Cell sorting was conducted using a FACSAria II cytometer (BD Biosciences). Data analysis was performed using FlowJo vX.10.8 software.

### Cell counting kit-8 assay

To evaluate the cytotoxicity of NK92MI cells, K562 cells were co-cultured with NK92MI cells at a 1:1 ratio in 96-well plates for various time points (0, 6, 12, 24, 36, and 48 hours). Subsequently, CCK-8 reagent (Biosharp, BS350A) was added to the wells according to the manufacturer’s instructions. Absorbance at 450 nm was then measured using a microplate reader (SpectraMax^®^ i3X).

### Adhesion assay

After different treatments, NK cells were harvested and purified using magnetic beads, and cell counts were determined. Subsequently, 1000 NK cells were seeded onto each 20mm slide (Biosharp, BS-20-RC) pre-coated with DSCs. After a 3-hour incubation, non-adherent NK cells were removed by washing, and the slides were fixed with 4% paraformaldehyde (Biosharp, BL539A) prior to immunofluorescence staining.

### Immunohistochemistry and immunofluorescence

Paraffin-embedded endometrium and decidual tissue sections were prepared. For immunohistochemistry, sections were incubated overnight at 4 °C in a humid chamber with either HA-binding protein (HABP) antibody (1:1000; Affinity, AF9083) or HAS2 antibody (1:1000; Invitrogen, PA5-25593). After three washes with PBS (Biosharp, BL302A), sections were incubated with a secondary antibody, and signals were visualized using a DAB Kit (ZSGB-Bio, ZLI-9017). Sections were then counterstained with hematoxylin (Biosharp, BL702B).

For immunofluorescence, cell-attached slides were incubated overnight at 4 °C in a humid chamber with Vimentin (1:500; Boster, BM4029) and CD56 antibody (1:1000; Proteintech, 14255-1-AP). Following PBS washes, slides were incubated with Donkey anti-mouse IgG 488 (1:500; Abcam, AB150105) and Goat anti-rabbit IgG 594 (1:500; Bioss, BS-0295G-BF594). Nuclei were counterstained with 4′,6-diamidino-2-phenylindole (DAPI; Biosharp, BL105A). Finally, all sections and slides were examined using a fluorescence microscope (Olympus, BX53).

### Modified Bitter-Muir method detected the concentration of HA

HA concentration in cell supernatants was determined using the modified BM method (31). Briefly, 5 mL of borax-sulfuric acid solution, pre-cooled to 4 °C in an ice bath, was mixed with 1 mL of either ultrapure water (for blank) or 1 mL of reference solutions of varying concentrations. After thorough mixing, the solutions were boiled for 10 minutes, then cooled to room temperature. Subsequently, 0.2 mL of carbazole reagent was added, and the mixtures were boiled for another 15 minutes before cooling to room temperature. Absorbance was measured at 530 nm using an ultraviolet spectrophotometer. A standard curve was generated from the reference solutions, enabling the calculation of HA concentration in the samples.

### Real time-qPCR

Total RNA was extracted using TRIzol^®^ reagent (Thermo, 15596026CN) and subsequently reverse-transcribed into cDNA using the HIScript III RT qPCR kit (Vazyme, R323-01). RT-qPCR was performed with Taq Universal SYBR Mix (Vazyme, Q712-02) on a qPCR instrument (Analytik Jena, qTOWER³). Transcriptional expression fold changes were calculated using the 2^−ΔΔCt^ method, with each sample analyzed in triplicate. Relative mRNA expression levels were normalized to housekeeping genes. Primer sequences for the genes of interest are provided in **Supplementary Table 3**. Melting curve analysis was conducted at the end of the reaction to avoid false-positive signals. In addition, the PCR products were checked for correct size by agarose gel electrophoresis. This experiment was conducted in accordance with the MIQE guidelines.

### Western blot analysis

DSCs were lysed in a buffer containing 50 mM Tris-HCl (pH 8.0), 150 mM NaCl, 1% Triton X-100 (v/v), and a protease inhibitor mixture (Thermo Scientific, A32965). Proteins from both conditioned medium and cell lysates were mixed with sample loading buffer, either with or without (non-reducing) TCEP (NCM Biotech), and denatured by heating at 100 °C for 5 minutes. Protein concentrations were quantified using the BCA protein assay kit (Vazyme, E112-02). Twenty-five micrograms of protein were separated by SDS-PAGE (Vazyme, E304-01) using a Mini-Protein III system (Bio-Rad, 1658033). Separated proteins were then transferred to PVDF membranes (Millipore, ISEQ00010) for 90 minutes, followed by incubation in 5% skimmed milk in PBST (Beyotime, P0222) at room temperature for 1 hour to block non-specific binding. The PVDF membranes were incubated overnight at 4 °C with primary antibodies against HAS2 (1:1000; 63kD; Santa Cruz, sc-514737), FOSL2 (1:1000; 45kD; Proteintech, M02615-2), Wnt1 (1:500; 45kD; Boster, PB0408), β-catenin (1:500; 92kD; CST, 8480T) and GAPDH (1:5000; 36kD; Proteintech, HRP-60004). After three washes with 1× TBST solution (Biosharp, BL2351A), membranes were incubated at room temperature for 1 hour with peroxidase-conjugated goat anti-mouse IgG secondary antibody (Proteintech, SA00001-1). Following three additional washes, protein bands were visualized by chemiluminescence using the Immobilon Western Chemiluminescent HRP Substrate Kit (Vazyme, E411-04/05).

### ScRNA-seq data acquisition and quality control

The scRNA-seq dataset E-MTAB-6678 (decidual tissues from 11 normal samples) (18) and GSE214607 (decidual tissues from 5 normal and 3 recurrent spontaneous abortion (RSA) samples) (32) was analyzed using the Seurat (33) and harmony R packages (version 1.2.3) (34). Quality control was performed to exclude cells with < 200 or >10000 detected genes, mitochondrial gene expression exceeding 20%. The data were normalized using the LogNormalize method, and dimensionality reduction was conducted based on the top 3000 highly variable genes. Batch effects were corrected during PCA using the harmony algorithm, and the top 15 principal components were selected for clustering. To ensure the accuracy of cell annotation, the “FindMarkers” function was employed to identify genes preferentially expressed in each cluster and differentially expressed genes (DEG) between fibrotic and normal cells. Annotation of major cell clusters was performed using known cell type marker genes. Cluster-specific marker genes were identified using thresholds of |log2FC| > 1 and adjusted p-value < 0.05. Additionally, the abundance of cell types was visualized using the “ggplot2” and “ComplexHeatmap” packages (version 3.5.2/2.18.0) (35, 36).

### Statistical analyses

All statistical analyses were performed using GraphPad Prism 9. For comparisons between two groups, either a paired or an unpaired t-test was employed. When data did not meet assumptions for parametric tests (normal distribution and variance homogeneity), the Wilcoxon matched-pairs signed-rank test or a Mann-Whitney U test was used, respectively. For comparisons among multiple groups, a one-way ANOVA test was utilized. If parametric assumptions were not met, a Kruskal-Wallis test was applied. Each experiment was independently repeated three times. Data are presented as mean ± standard deviation (SD) for normally distributed data, or as median and quartiles for non-normally distributed data. A *P*-value of less than 0.05 was considered statistically significant.

## Results

### DSCs enhance the tissue-resident capacity of peripheral–derived NK cells

**Figure 1:**
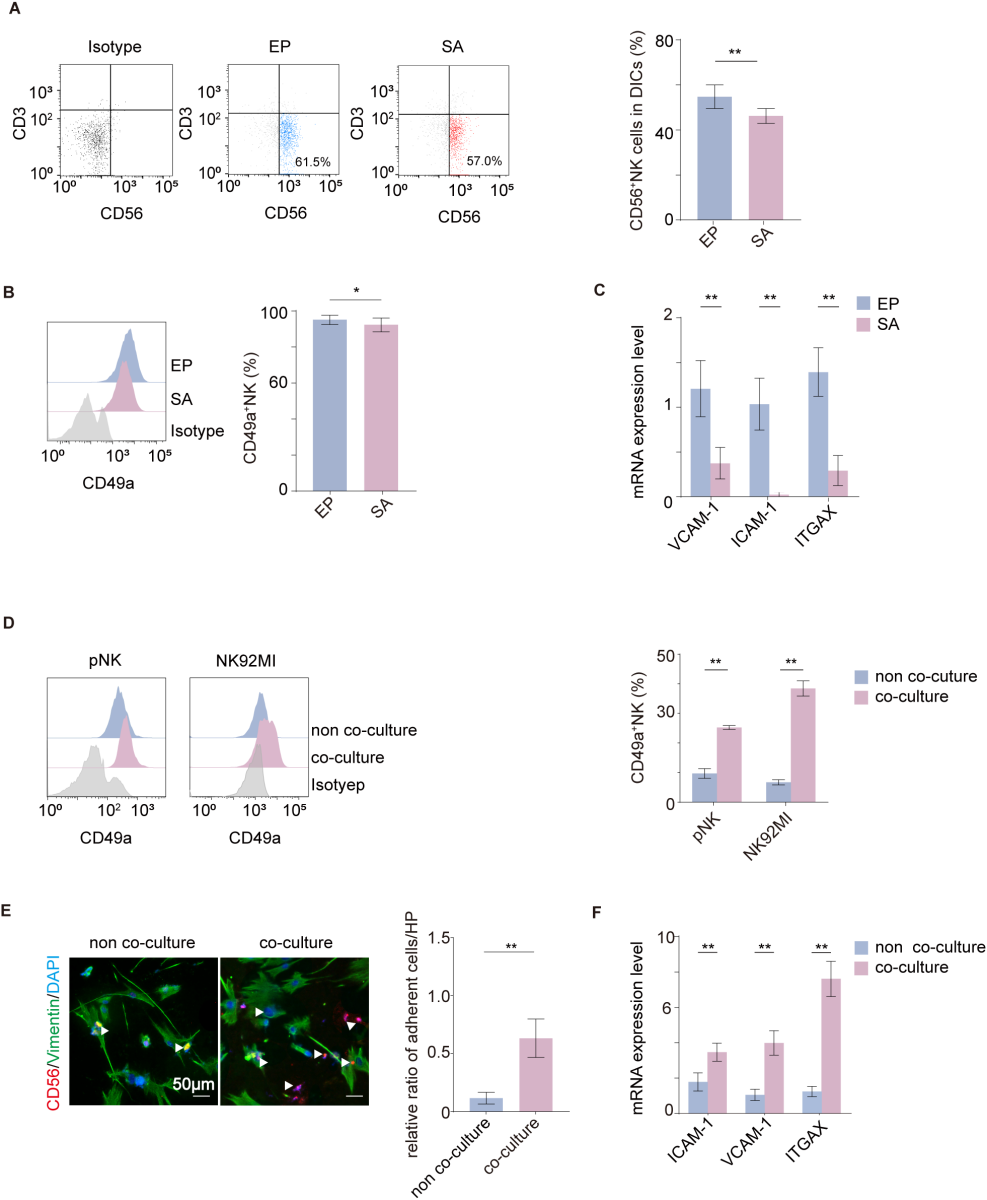
Decidual stromal cells enhanced tissue residency of peripheral-derived NK cells. **(A)** dNK cell proportion assessed by flow cytometry in normal pregnancies (*n* = 19) and spontaneous abortion (SA) cases (*n* = 19). **(B)** CD49a expression levels in dNK cells from EP and SA cases (*n* = 19 per group) **(C)** Adhesion molecules (*ICAM-1, VCAM-1, ITGAX*) measured by RT-qPCR in dNK cells from normal pregnancies and SA (*n* = 10 per group). **(D)** The expression of CD49a by flow cytometry in pNK and NK92MI cells after co-culture with DSCs (*n* = 6 per group). **(E)** Immunofluorescence analysis of the adhesive capacity of recovered NK92MI cells to DSC-coated coverslips with or without prior DSC co-culture (scale bars, 50 μm)(*n* = 9 per group). **(F)** The expression of adhesion molecules (*ICAM-1, VCAM-1, and ITGAX*) in NK cells assessed by RT-qPCR after co-culture with PBS (*n* = 6) or DSCs (*n* = 6). Data are expressed as mean ± SD; *P < 0.05; **P < 0.01.

Flow cytometry (gating strategy, **Supplementary Figure 1A**) revealed a significantly lower proportion of dNK cells in the SA group compared to the normal pregnancy group (**Figure 1A**). Additionally, flow cytometric analysis of the tissue-resident marker CD49a (**Figure 1B**) and RT-qPCR quantification of adhesion molecules (*ICAM-1*, *VCAM-1*, and *ITGAX*, **Figure 1C**) revealed a significant downregulation in the NK cells of the SA group. These findings suggest that impaired NK cell residency is associated with SA.

CD49a distinguishes tissue-resident dNK cells (15, 37), consistent with the more adhesion molecules expressed on dNK cells than those on pNK cells (**Supplementary Figure 1C**). Co-culture with DSCs induced a tissue-resident phenotype in peripheral-derived NK cells. This was evidenced by a significant upregulation of CD49a, a canonical dNK marker, in both pNK and NK92MI cells (**Figure 1D**). Given this consistent observation across both cell types, NK92MI cells were subsequently selected to represent primary pNK cells for further validation experiments. Furthermore, cell residency (**Figure 1E**), and expression of adhesion molecules (*ICAM-1, VCAM-1* and *ITGAX*) of NK92MI cells (**Figure 1F**) were enhanced after co-culture. These results demonstrate that the tissue-resident capacity of NK92MI cells is enhanced following co-culture with DSCs.

### DSC-derived HA enhances the tissue residency of NK92MI cells

**Figure 2:**
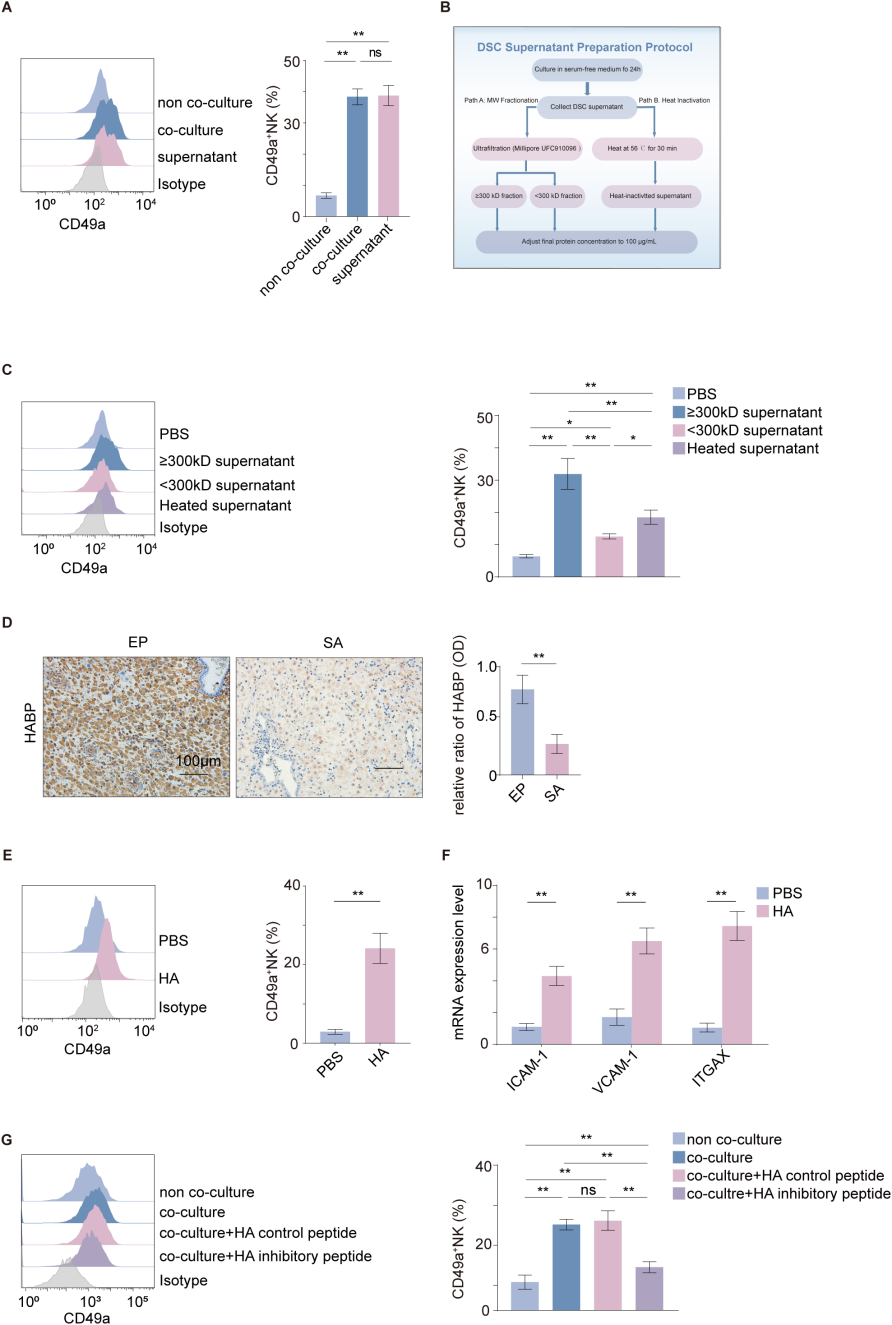
DSC-derived hyaluronic acid enhanced NK92MI tissue residency. **(A)** CD49a expression in NK92MI cells after co-culture with DSCs or treatment with DSC supernatant (*n* = 6 per group). **(B)** Schematic workflow of supernatant processing and preparation. **(C)** CD49a expression in NK92MI cells following treatment with three types of DSC supernatants: ≥300 kD, <300 kD, and heat-inactivated protein fractions (*n* = 5 per group). **(D)** The expression of HABP in human decidual tissue (scale bars, 100 μm) detected by immunohistochemistry (*n* = 10 per group). **(E)** The expression of CD49a in NK92MI cells was detected after the addition of HMW-HA (*n* = 6 per group). **(F)** The expression of adhesion molecules (*ICAM-1*, *VCAM-1*, and *ITGAX*) in NK92MI cells assessed after the addition of HMW-HA (*n* = 6 per group). **(G)** CD49a expression levels were assessed in NK92MI cells cultured alone or co-cultured with DSCs, in the presence or absence of an HA inhibitory peptide or a control peptide (*n* = 6 per group). Data are expressed as mean ± SD; *P < 0.05; **P < 0.01; ns, not significant. EP, early pregnancy; SA, spontaneous abortion.

Treatment of NK92MI cells with supernatant from DSCs up-regulated CD49a expression, similar to direct co-culture, indicating the involvement of soluble factors in the conversion (**Figure 2A**). To identify the responsible factors, the supernatant was fractionated by molecular weight size. The effect was predominantly observed in the large-molecule (≥300 kD) fraction and was partially resistant to heat denaturation (**Figure 2B**), which suggests that the active component is a high-molecular-weight, non-protein substance (**Figure 2C**).

Furthermore, HA, a high-molecular-weight polysaccharide and a major component of the extracellular matrix, was subsequently considered a strong candidate. HA expression was significantly elevated in the decidua of normal pregnancies (**Figure 2D**) and in the supernatant from DSCs (**Supplementary Figure 2B**). Given that the maternal-fetal interface is predominantly characterized by HMW-HA during normal pregnancy (38, 39), supplementation with HMW-HA in the NK92MI cell line successfully upregulated the expression of CD49a (**Figure 2E**) and adhesion-related factors (**Figure 2F**). Furthermore, neutralization of HA in the culture supernatant via the addition of an HA inhibitory peptide resulted in significantly lower CD49a expression in co-cultured NK92MI cells compared to the control peptide group (**Figure 2G**). These results indicate that HA, particularly HMW-HA, is a key factor responsible for the enhanced tissue-resident capacity of NK92MI cells.

### HA/CD44 signaling axis mediates enhanced tissue residency of NK92MI cells

**Figure 3:**
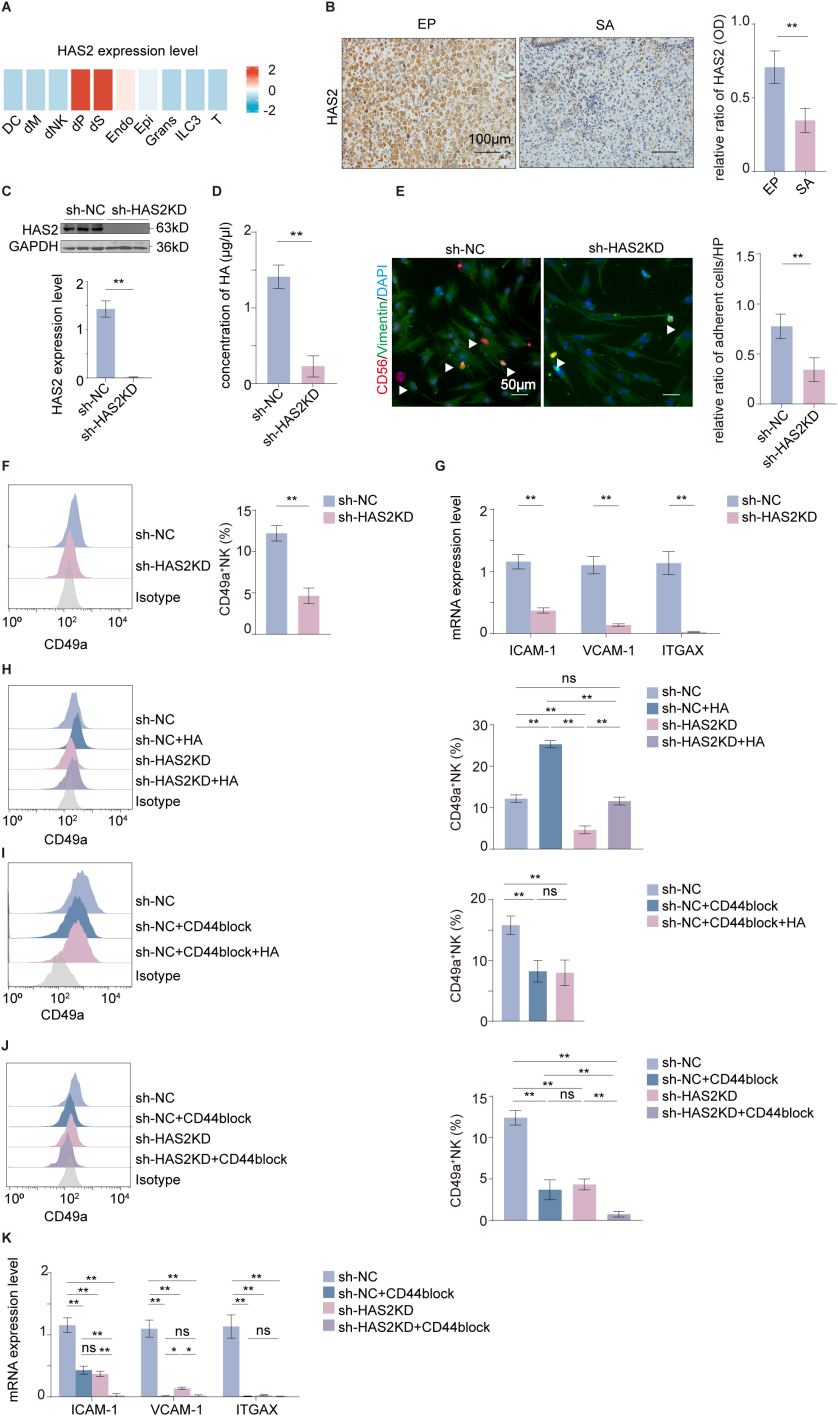
HA/CD44 axis promotes NK92MI tissue residency. **(A)** Analysis of HAS2 expression levels across different cell subpopulations in normal decidual tissue based on scRNA-seq data (*n* = 11). **(B)** The expression of HAS2 in human decidual tissue (scale bars, 100 μm) by immunohistochemistry (*n* = 10 per group). **(C)** Confirmation of HAS2KD in DSCs by western blot (*n* = 3 per group). **(D)** HA concentration in DSC supernatant (*n* = 6 per group). **(E)** The residency of NK92MI cells in DSCs (scale bars, 50 μm) after co-culture with HAS2KD DSCs (*n* = 9 per group). **(F, G)** The expression of CD49a and adhesion molecules (*ICAM-1, VCAM-1*, and *ITGAX*) in NK92MI cells after co-culture with HAS2KD DSCs (*n* = 6 per group). **(H)** CD49a expression in NK92MI cells co−cultured with control DSCs or HAS2KD DSCs, with or without exogenous HMW−HA (*n* = 6 per group). **(I)** CD49a expression in NK92MI cells from DSC-NK co-cultures treated with exogenous HMW-HA after CD44 blocking (*n* = 6 per group). **(J, K)** The expression of CD49a and adhesion molecules (*ICAM-1, VCAM-1*, and *ITGAX*) in NK92MI cells co−cultured with control DSCs or HAS2KD DSCs, with or without CD44−blocking antibody. Data are expressed as mean ± SD; * *P* < 0.05; **P < 0.01; ns, not significant. EP, early pregnancy; SA, spontaneous abortion.

The production of HA, especially HMW-HA, is largely mediated by HAS2 (38). In normal pregnancy, scRNA-seq analysis revealed that HAS2 is primarily expressed in DSCs and pericytes, with low expression levels observed in endothelial cells (**Figure 3A**) (18). Consistent with HA levels, expression of its synthesizing enzyme, HAS2, was significantly higher in the decidua of normal pregnancies than in SA (**Figure 3B**). Furthermore, HAS2 expression in DSCs was elevated compared to ESCs (**Supplementary Figure 2C**). We knocked down HAS2 in DSCs (**Figure 3C** and **Supplementary Figure 2D**), which did not increase the expression of other HAS family members (HAS1 and HAS3) (**Supplementary Figure 2E**), resulting in significantly reduced HA secretion (**Figure 3D**). Co-culture with these HAS2-deficient DSCs impaired the phenotypic transition of NK92MI cells, leading to diminished cell adhesion (**Figure 3E**), reduced CD49a expression (**Figure 3F** and **Supplementary Figure 2F**), and down regulated expression of adhesion molecules (**Figure 3G**). Crucially, this impairment was rescued by adding exogenous HMW-HA (**Figure 3H**), demonstrating that DSCs mediate NK92MI cell phenotypic transition primarily dependent on HAS2-mediated HA synthesis.

Since NK cells highly express CD44 (the primary receptor for HA), we employed a CD44-blocking antibody in the co-culture system. Notably, the reduction in CD49a expression induced by CD44 blockade could not be rescued by the addition of exogenous HMW-HA (**Figure 3I**), suggesting that the HMW-HA-mediated effect is strictly CD44-dependent. The results showed that the expression of CD49a (**Figure 3J**) and adhesion molecules (**Figure 3K**) were reduced, which was amplified when combined with HAS2KD in DSCs.

### DSCs reduce NK cell cytotoxicity by regulating the proportion of CD44^high^ and CD44^low^ subpopulations via the HA/CD44 axis

Studies have demonstrated that, in contrast to pNK cells, dNK cells primarily mediate immune tolerance rather than cytotoxic activity (17, 40). Furthermore, we identified two dNK cell subpopulations based on their CD44 expression: CD44^high^ and CD44^low^ (**Supplementary Figure 3A**). The CD44^high^ subpopulation was highly cytotoxic, evidenced by its significantly greater secretion of granzyme B (GZMB), tumor necrosis factor-α (TNF-α), and interferon-γ (IFN-γ) (**Supplementary Figure 3B**). Consistent with dNK cells, this phenomenon was also evident in the NK92MI cell line (**Supplementary Figures 3C, D**).

**Figure 4:**
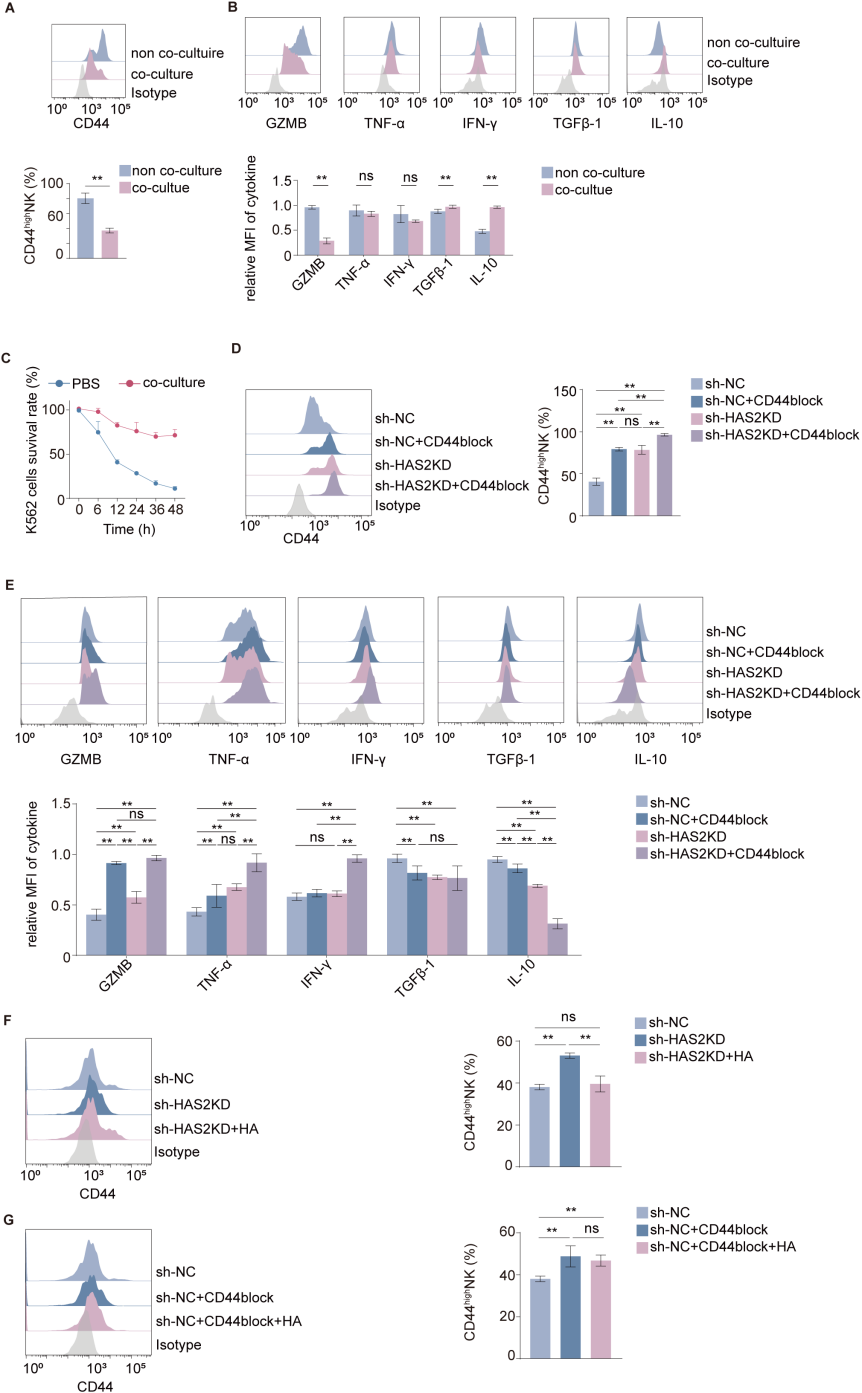
DSCs ameliorate NK92MI cell cytotoxicity by modulating CD44 subpopulations via the HA/CD44 axis. **(A)** The proportion of the CD44^high^ subpopulation in NK92MI cells after co-culture with DSCs (*n* = 6 per group). **(B)** The expression of cytokines (GZMB, TNF-α, IFN-γ, TGF-β1 and IL-10) in NK92MI cells detected by flow cytometry after co-culture with DSCs (*n* = 5 per group). **(C)** The viability of K562 cells determined after exposure to NK cells previously co-cultured with DSCs (*n* = 6 per group). **(D, E)** The proportion of the CD44^high^ subpopulation and the expression of effector molecules (GZMB, TNF−α, IFN−γ, TGF−β1 and IL−10) in NK92MI cells co−cultured with control DSCs or HAS2KD DSCs, with or without CD44−blocking antibody (*n* = 6 per group). **(F, G)** The proportion of the CD44^high^ subpopulation in NK92MI cells co−cultured with control DSCs, HAS2KD DSCs, or DSCs plus CD44−blocking antibody after the addition of exogenous HMW−HA (*n* = 5 per group). Data are expressed as mean ± SD; **P < 0.01; ns, not significant.

Co-culture with DSCs reduced the proportion of the highly cytotoxic CD44^high^ NK92MI cell subpopulation (**Figure 4A**). This phenomenon was accompanied by a shift of cytokine profile, with the up-regulation of immune-tolerant cytokines such as transforming growth factor-β1 (TGF-β1) and interleukin-10 (IL-10), and the down-regulation of cytotoxic factors, which was confirmed at both mRNA and protein levels (**Figure 4B**). A CCK-8 assay subsequently confirmed a significant reduction in the overall cytotoxicity of NK92MI cells after co-culture (**Figure 4C**).

Either co-culturing NK92MI cells with HAS2KD DSCs or blocking the CD44 receptor on NK cells independently led to an increased proportion of the cytotoxic CD44^high^ subpopulation (**Figure 4D**) and promoted a pro-inflammatory cytokine profile (**Figure 4E**). These effects were aggravated when both conditions were applied simultaneously. Notably, the increase in the cytotoxic CD44^high^ NK92MI subpopulation induced by HAS2 knockdown in DSCs was effectively rescued by exogenous HMW-HA treatment (**Figure 4F**). However, the CD44 blockade–induced increase in this cytotoxic subpopulation was not reversed by HMW-HA supplementation. (**Figure 4G**).

### Dynamics of dNK cell subsets support pregnancy maintenance via the regulation of CD44^high^ and CD44^low^ subpopulations

**Figure 5:**
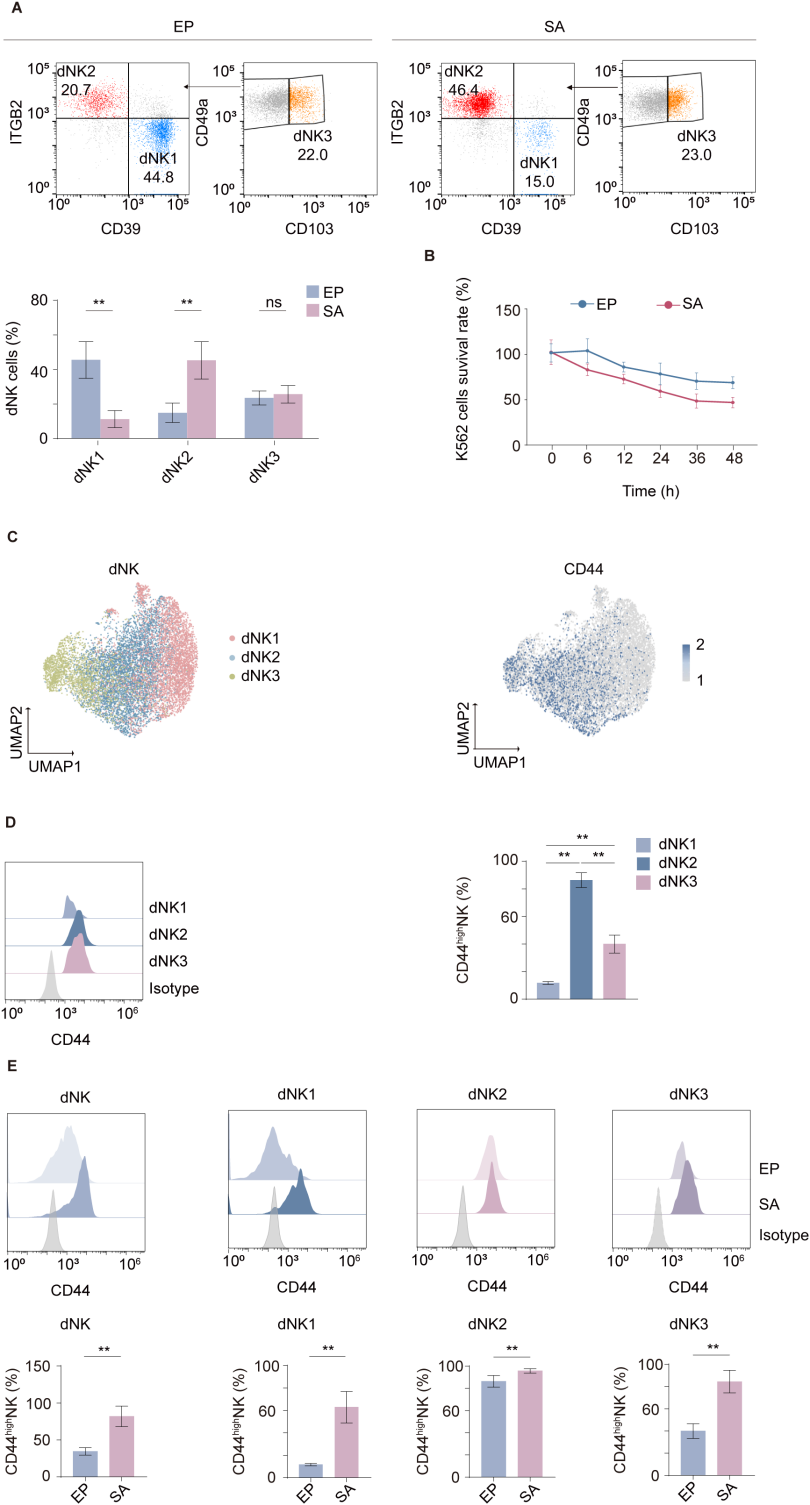
DSCs ameliorate NK cell cytotoxicity by regulating the proportion of CD44^high^ and CD44^low^ subpopulations via the HA/CD44 axis. **(A)** The proportion of dNK1, dNK2, and dNK3 subpopulations in dNK cells from individuals with normal pregnancies and SA (*n* = 19 per group). **(B)** The viability of K562 cells after co-culture with total dNK cells from individuals of normal pregnancies and SA (*n* = 9 per group). **(C)** UMAP plot of scRNA-seq data from normal decidual tissues showing elevated CD44 expression in the dNK2 and dNK3 subpopulation (*n* = 11). **(D)** The proportion of the CD44^high^ subpopulation within the three subpopulations in individuals with normal pregnancies (*n* = 6). **(E)** The proportion of the CD44^high^ subpopulation in total dNK cells and the three subpopulations between individuals from normal pregnancies and SA (*n* = 6 per group). Data are expressed as mean ± SD; **P < 0.01; ns, not significant. EP, early pregnancy; SA, spontaneous abortion.

dNK cells were categorized into dNK1, dNK2, and dNK3 subgroups. SA patients showed a lower proportion of the dNK1 subgroup and higher proportions of the dNK2 and dNK3 subgroups than normal pregnant women (**Figure 5A**). Compared to normal pregnancies, dNK cells from patients with SA exhibited higher cytotoxicity (**Figure 5B**). This heightened cytotoxicity is driven by a significant shift in dNK subpopulations. ScRNA-seq analysis of samples from normal pregnancies indicated that CD44 is predominantly expressed in the dNK2 and dNK3 subsets (**Figure 5C**). Consistent with these findings, flow cytometry analysis also demonstrated that the dNK1 subgroup contained the lowest proportion of highly cytotoxic CD44^high^ cells compared to the dNK2 and dNK3 subgroups (**Figure 5D**). Furthermore, the proportion of these CD44^high^ cells was significantly higher in the SA group compared to normal pregnancies both in total dNK cells and within individual subgroups (**Figure 5E**). These observations suggested that the imbalance of dNK1, dNK2 and dNK3 subpopulations in SA contributes to an aberrant increase in cytotoxic CD44^high^ cells.

### DSC-derived HA drives the enrichment of the dNK1-like subset

**Figure 6:**
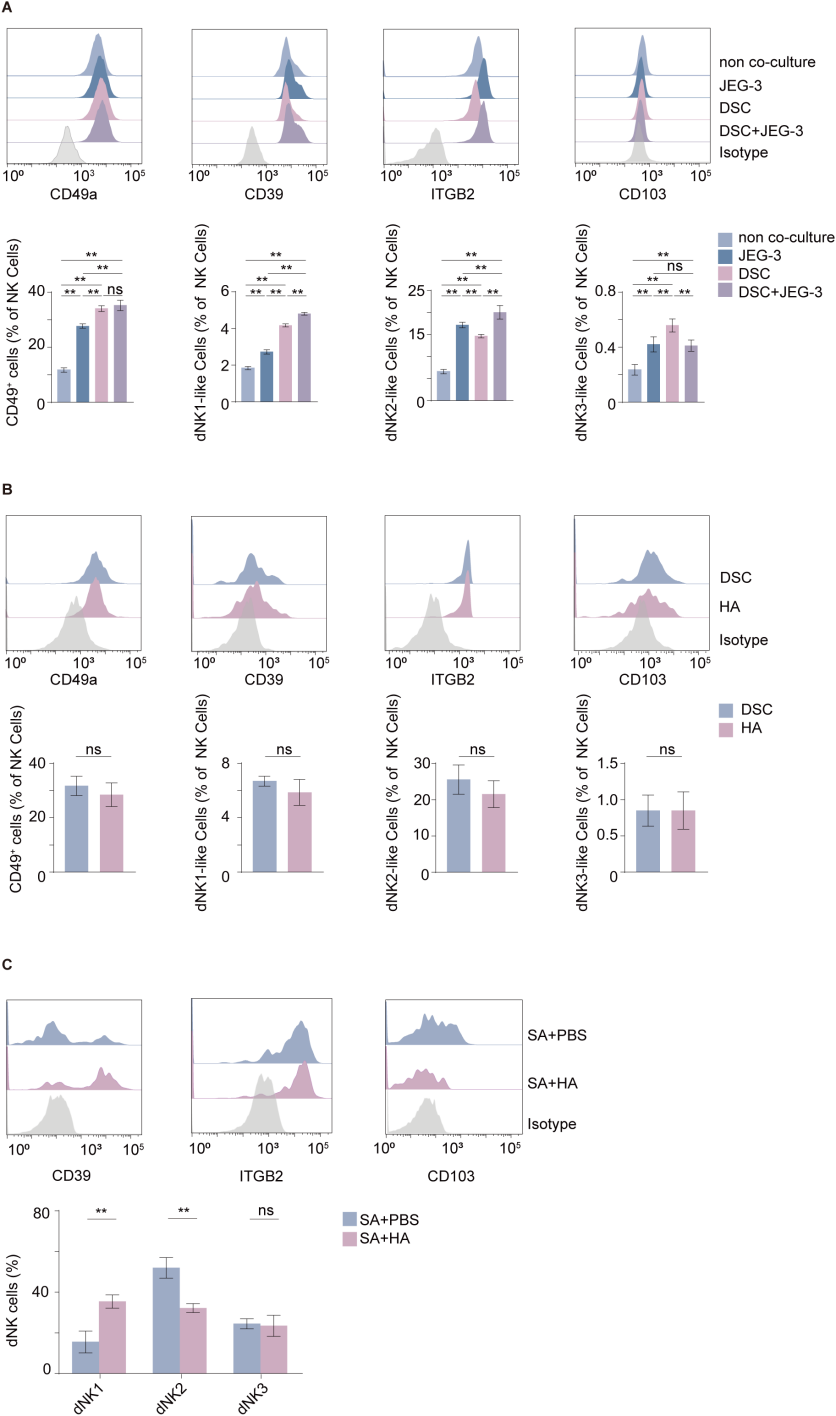
DSC-derived HA promotes the enrichment of dNK1-like subsets. **(A)** Distribution of dNK-like and dNK1/2/3-like subpopulations in control NK92MI cells and in NK92MI cells after co-culture with JEG-3 cells, DSCs, or DSCs plus JEG-3 cells (*n* = 6 per group). Right panel shows the relative proportions of the three dNK1/2/3-like subpopulations under the indicated co-culture conditions (*n* = 5 per group). **(B)** Proportions of dNK-like and dNK1/2/3-like subpopulations in NK92MI cells following exposure to HMW-HA or co-culture with DSCs (*n* = 6 per group). **(C)** Proportions of dNK1/2/3-like subpopulations in primary dNK cells from patients with SA after exposure to HMW-HA (*n* = 5 per group). Data are expressed as mean ± SD; **P < 0.01; ns, not significant. dNK1-like: CD49a^+^CD39^+^ITGB2^-^CD103^-^ NK cells; dNK2-like: CD49a^+^CD39^-^ITGB2^+^CD103^-^ NK cells; dNK3-like: CD49a^+^CD103^+^ NK cells. EP, early pregnancy; SA, spontaneous abortion.

Compared with monoculture conditions or co-culture with JEG3 cells alone, the presence of DSCs markedly promoted the enrichment of the dNK1-like subset in peripheral-derived NK92MI cells (**Figure 6A**). Notably, treatment with exogenous HMW-HA alone largely recapitulated this effect (**Figure 6B**). Moreover, HMW-HA treatment was associated with an increased proportion of dNK1 cells and a concomitant decrease in dNK2 cells within dNK populations from patients with SA (**Figure 6C**). Together, these results indicate that DSCs, through HMW-HA/CD44 crosstalk, led to a relative enrichment of the dNK1-like subset.

### HA promotes the acquisition of dNK1-like phenotypic features in NK92MI cells via Wnt–FOSL2 signaling

**Figure 7:**
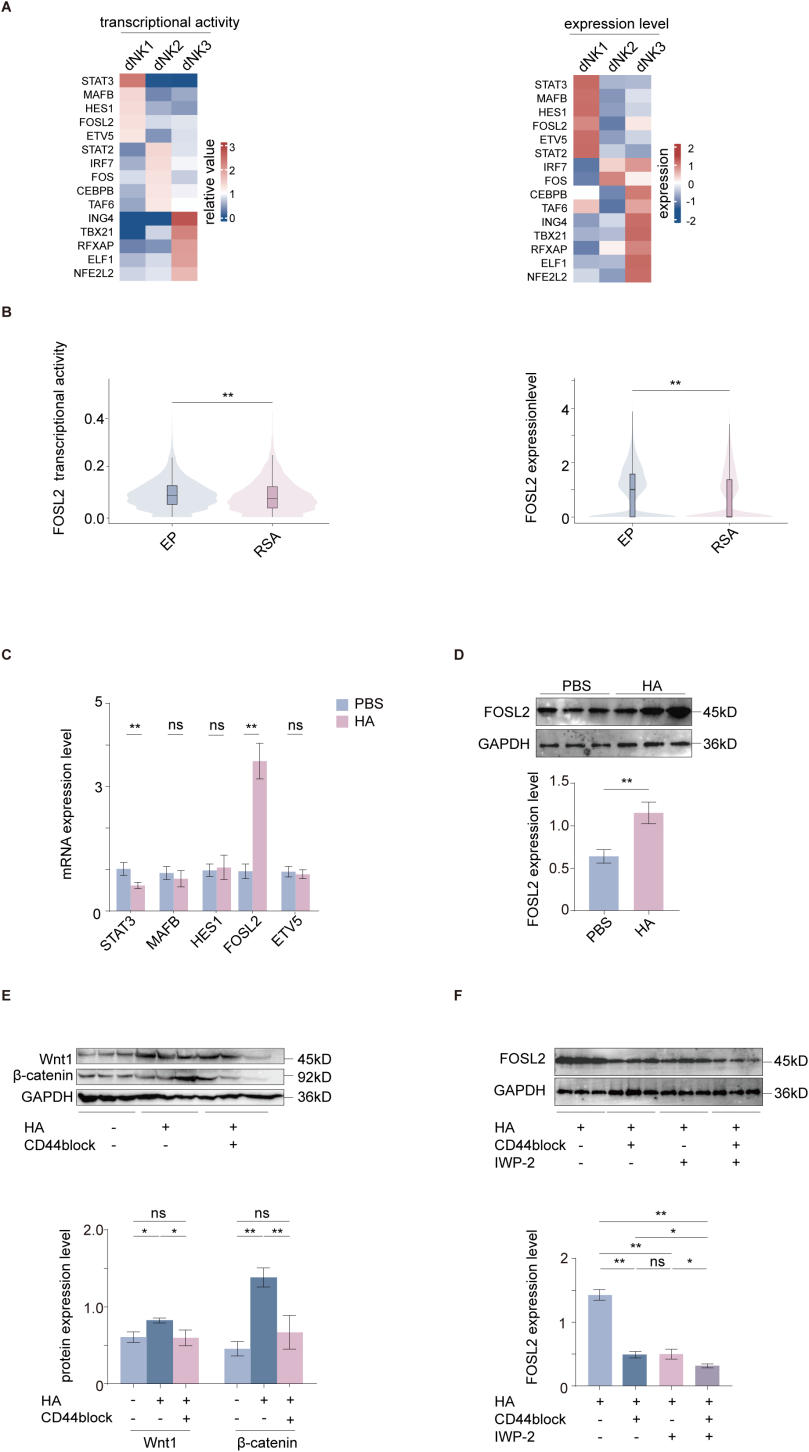
HA-induced activation of the canonical Wnt pathway upregulates FOSL2 to expand the dNK1-like subpopulation. **(A)** Analysis of transcriptional activity and expression levels of transcription factors in three dNK subsets using scRNA-seq data from normal decidual tissue (*n* = 11). **(B)** Transcriptional activity and expression levels of *FOSL2* in decidual tissues from normal pregnancy (*n* = 5) and RSA groups (*n* = 3). **(C)** Changes in NK92MI cells of the five transcription factors (*STAT3, MAFB, HES1, FOSL2, ETV5*) characterized by high transcriptional activity and expression in the dNK1 subset following HMW-HA treatment, as determined by RT−qPCR (*n* = 6 per group). **(D)** *FOSL2* expression in NK92MI cells following HMW-HA treatment, assessed by WB (*n* = 3 per group). **(E)** Wnt1 and β-catenin protein expression in NK92MI cells treated with or without HMW-HA in the presence or absence of CD44 blockade (*n* = 3 per group). **(F)** *FOSL2* expression in HA−treated NK92MI cells after addition of CD44−blocking antibody or the Wnt pathway inhibitor IWP−2 to the culture system (*n* = 3 per group). Data are expressed as mean ± SD; *P < 0.05; **P < 0.01; ns, not significant. EP, early pregnancy; SA, spontaneous abortion; RSA, recurrent spontaneous abortion.

We analyzed specific transcription factors (TFs) in the dNK1, dNK2, and dNK3 subsets in the decidua of normal pregnancies using a public dataset (**Figure 7A**) (18). Among the transcription factors specifically enriched in the dNK1 subset, *FOSL2* has been reported to participate in the transition from the dNK2 to the dNK1 subset (41). Moreover, *FOSL2* transcriptional activity and expression were significantly higher in dNK cells from normal pregnancies compared to those from patients with RSA (**Figure 7B**) (32).

**Figure 8:**
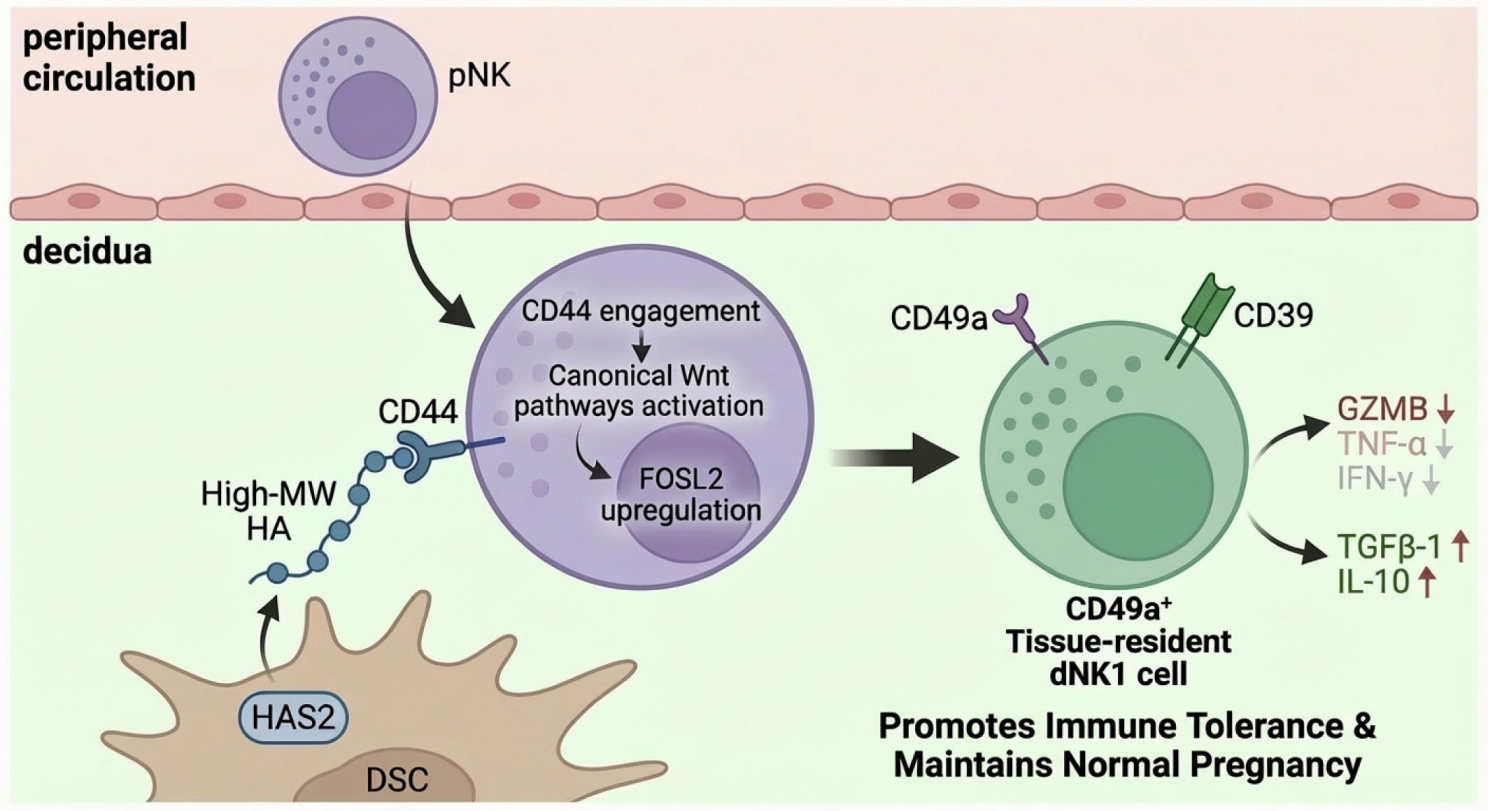
Schematic diagram of this study.

Analysis of five dNK1−specific TFs with high expression levels—*STAT3*, *HES1*, *FOSL2*, *MAFB* and *ETV5. FOSL2* emerged as the most notably upregulated candidate after HMW-HA treatment (**Figure 7C**). Consistent with the mRNA levels, subsequent protein-level analysis confirmed that HMW-HA treatment significantly enhanced *FOSL2* expression in NK92MI cells (**Figure 7D**). Moreover, *FOSL2* is a downstream target of the canonical Wnt signaling pathway (42), subsequent WB analysis verified that HMW-HA treatment enhanced expression of Wnt1 and β−catenin, two key components of the canonical Wnt pathway (**Figure 7E**). Indeed, blocking the HA receptor CD44 or treating cells with IWP−2—an inhibitor of the canonical Wnt pathway—led to a marked reduction in *FOSL2* expression (**Figure 7F**). These findings suggest that the interaction between HMW-HA and CD44 on the NK cell surface activates the canonical Wnt signaling pathway, which subsequently upregulates *FOSL2* expression. Based on these findings, a schematic model is proposed in **Figure 8**.

## Discussion

SA is a common early pregnancy complication with a complex etiology, including fetal genetics, maternal anatomy, endocrine disorders, autoimmune diseases, infections, poor health habits, and so forth. Maintaining immune homeostasis at the maternal-fetal interface is crucial for successful pregnancy while abnormal immunity is considered the major cause of unexplained SA. NK cells are the main immune cells at this interface, and their abundance and function shape the local immune environment. In early pregnancy, a subset of pNK cells migrates to the maternal-fetal interface and is transformed into dNK cells with enhanced residency and reduced cytotoxicity (19–23). In the present study, we elucidated a novel mechanism underlying this transition. Our findings demonstrated that DSCs secrete a high-molecular-weight glycosaminoglycan that actively mediates the phenotypic and functional transformation of pNK cells into the dNK-like cell lineage.

As a high-molecular-weight polysaccharide, HA is a pivotal constituent of the extracellular matrix, involved in diverse physiological processes including cell migration (43), cell proliferation (44), wound healing (45), angiogenesis (46), tumor invasion and metastasis (47). While its involvement in cardiovascular and cerebrovascular systems has been extensively explored (48, 49), emerging evidence in reproductive biology highlights its importance in oocyte maturation, embryo implantation and development (50–53). The function of HA is dictated by its molecular weight: HMW-HA (500–20,000 kD) is anti-inflammatory, while low-molecular-weight HA (LMW-HA; 3–40 kD) is pro-inflammatory. This immunomodulatory role is crucial for pregnancy, as our prior investigations showed that HMW-HA levels were elevated during normal gestation but were diminished in RSA (38). We have previously demonstrated that DSCs synthesized HMW-HA predominantly via the enzyme HAS2, then engaged CD44 receptors on both DSCs and EVTs to positively regulate their biological functions (38, 54). Given that NK cells also highly expressed CD44, the present study demonstrated that their functional transformation is similarly dependent on this DSC-driven HA/CD44 axis.

It is established that the HA/CD44 axis exerts immunomodulatory control by reducing cell cytotoxicity and promoting immune tolerance, such as inducing M2 macrophage polarization and enhancing the function of regulatory T cells (30, 55–57). Structural studies have shown that the binding of HA to CD44 can induce a transition of CD44 from a low-affinity to a high-affinity state, accompanied by localized conformational rearrangements and dynamic changes within its HA-binding domain, thereby initiating downstream signal transduction (58, 59). Notably, HMW-HA may engage CD44 in a sustained or multivalent manner and is thought to promote receptor clustering or spatial reorganization at the cell surface. On this basis, HMW-HA may preferentially interact with cell subsets expressing high levels of CD44 and modulate receptor organization or epitope accessibility, ultimately leading to a reduced proportion of CD44^high^ cells. Moreover, dNK cells show higher cytotoxicity in SA than in normal pregnancy. This difference is linked to disparities among dNK subpopulations. ScRNA-seq has classified dNK cells into three main subgroups (dNK1, dNK2, dNK3) and a smaller, highly proliferative dNKp subset (18, 57, 60). The three major subgroups all express CD49a and CD9. dNK1 expresses CD39 and high levels of KIRs, involved in EVT recognition. The dNK2 subset is characterized by ANXA1 and ITGB2 expression and participates in the recruitment of EVTs and dendritic cells. dNK3 is comparatively less abundant, with marker expression of CD160, KLRB1, and CD103 (18). The relative proportions of these three dNK subsets are intrinsically linked to the clinical outcome of pregnancy. It was demonstrated that the proportion of the dNK1 subpopulation is significantly reduced in patients with RSA compared to those with ongoing pregnancies (61). In this study, we observed a similar reduction in the dNK1 subpopulation in SA patients. Our analysis revealed that cytotoxicity is primarily associated with a CD44^high^ dNK cell population. Further characterization demonstrated that the dNK1 subpopulation is distinguished by a significantly lower proportion of these CD44^high^ cells, thereby accounting for its intrinsically reduced cytotoxic profile. Subsequently, we found that co-culture with DSCs significantly promoted the differentiation of NK92MI cells toward the dNK1-like phenotype. From a mechanistic perspective, this transition is mediated by the elevation of *FOSL2* induced by HA-CD44 binding. *FOSL2* is characterized as a negative regulator of NK cell development (62) and a dNK1-specific transcription factor, exhibiting markedly higher expression in dNK1 cells than in dNK2 or dNK3 populations (41). It functions by regulating specific dNK1 protein profiles and assisting IL-15 signaling to drive the dNK2-to-dNK1 transition (41). Regarding its upstream regulation, studies in macrophages have shown that *FOSL2* is a target of the canonical Wnt pathway (42). Our findings confirm that this phenomenon is not limited to macrophages but also occurs in NK cells. Furthermore, *in vitro* treatment of dNK cells isolated from SA patients with HA sufficiently restored the proportion of the dNK1-like subpopulation, further confirming HMW-HA as a key factor mediating this beneficial transformation.

Notably, the functional role of dNK cells, particularly the dNK1 subset, is highly dependent on the prevailing maternal immune status. Consistent with numerous other studies, our research utilized samples from a population with unexplained SA, in which individuals with autoimmune diseases or immune stress states resulting from infection were excluded. Consequently, a reduction in the dNK1 cell population is correlated with pathologies linked to EVTs dysfunction, including SA, preeclampsia, and fetal growth restriction. However, in states of autoimmune diseases or immune activation, the dNK cell population, and specifically the dNK1 subset, can become activated and consequently reprogrammed to adopt a cytotoxic phenotype. For instance, during immune activation in mouse models, trNK cells—the murine counterpart to human dNK1 cells—have been shown to proliferate and upregulate GZMB expression, leading to neurodevelopmental abnormalities in offspring (63, 64). Similarly, in patients with obstetric antiphospholipid syndrome, an expansion of LILRB1^+^ dNK cells (predominantly dNK1) was observed, which coincided with reduced expression of immune inhibitory receptors and a heightened cytotoxic profile (65).

This study elucidated a critical regulatory mechanism by which DSCs orchestrate maternal-fetal immune tolerance through their interaction with dNK cells. We demonstrated that DSCs ameliorate dNK cell cytotoxicity by adjusting the proportion of the highly cytotoxic CD44^high^ subpopulation, a process fundamentally mediated by the HA/CD44 axis. Specifically, the dNK1 subpopulation exhibits a significantly lower proportion of CD44^high^ cells, thereby contributing to its intrinsically reduced cytotoxic profile. Particularly, our work highlighted that DSCs effectively precondition dNK-like cells, guiding their differentiation towards the dNK1-like phenotype to foster a favorable microenvironment for interaction with EVTs.

However, several limitations of the present study should be acknowledged. Our findings are primarily based on analyses of human decidual tissue samples and *in vitro* cellular experiments, which cannot fully recapitulate the complex microenvironment of the maternal-fetal interface *in vivo*. Therefore, further validation using appropriate mouse models will be necessary in future studies. For instance, miscarriage models or HAS2 cKO mice could be established to clarify the impact of HA deficiency on NK cell phenotype and function *in vivo*. In addition, NK cells pretreated with HA *in vitro* could be adoptively transferred into miscarriage or HAS2 cKO mouse models to evaluate pregnancy outcomes. Such approaches would provide more direct evidence for the role of HA-mediated regulation of NK cell phenotype and function in the maintenance of normal pregnancy.

In summary, our study delineated a novel communication of DSCs with NK cells, highlighting the pivotal role of the HA/CD44 axis in shaping the dNK cell landscape to maintain immune homeostasis at the maternal-fetal interface. While the specific downstream effects on EVT function warrant further investigation, these insights not only deepen our understanding of pregnancy immunology but also offer promising interventions to prevent and treat pregnancy complications associated with abnormal immunity.

## Data Availability

The raw data supporting the conclusions of this article will be made available by the authors, without undue reservation.

## Supplementary material

Supplementary Figure 1 Isolation and characterization of dNK and pNK cells and analysis of adhesion molecule expression. **(A)** Flow cytometry gating strategy within dNK. **(B)** The purity of isolated dNK and pNK cells assessed by flow cytometry (*n* = 6 per group). **(C)** Adhesion molecule (*ICAM-1, VCAM-1* and *ITGAX*) mRNA expression in dNK and pNK cells (*n* = 10 per group). Data: mean ± SD; **P* < 0.05, ***P* < 0.01, ns, not significant.

Supplementary Figure 2 Validation of HAS2 knockdown in DSCs and its effect on NK cell CD49a expression. **(A)** Prolactin expression in ESCs after decidualization treatment by RT-qPCR (n=6 per group). **(B)** HA concentration in DSC supernatant. **(C)** HAS2 expression in ESCs and DSCs (*n* = 6 per group). **(D)** Efficacy of two plasmids for the construction of DSCs with HAS2KD (*n* = 6 per group). **(E)** Expression of HAS1 and HAS3 in DSCs with HAS2KD (*n* = 6 per group) **(F)** CD49a expression in NK cells co−cultured with control DSCs or HAS2KD−2 DSCs (*n* = 6 per group). Data: mean ± SD; **P* < 0.05, ***P* < 0.01, ns, not significant.

Supplementary Figure 3 CD44^high^ and CD44^low^ subpopulation definition and effector molecule profiles in dNK and NK92MI cells. **(A)** Division of dNK cells into CD44^high^ and CD44^low^ subpopulation. **(B)** Effect molecules (GZMB, TNF-α, IFN-γ, TGF-β1, IL-10) expression in CD44^high^ and CD44^low^ dNK cells by flow cytometry (*n* = 8 per group). **(C)** Division of NK92MI cells into CD44^high^ and CD44^low^ subpopulation. **(D)** Effect molecules (GZMB, TNF-α, IFN-γ, TGF-β1, IL-10) expression in CD44^high^ and CD44^low^ NK92MI cells by flow cytometry (*n* = 6 per group). Data are expressed as mean ± SD; **P* < 0.05; ***P* < 0.01; ns, not significant.
