# Supplementary figures and images for: Hyaluronic acid−CD44 signaling from decidual stromal cells orchestrates dNK1 differentiation and immune tolerance in early pregnancy
Source: Front Immunol. 2026 Mar 25;17:1777567. doi: 10.3389/fimmu.2026.1777567 (PMC13057500; doi:10.3389/fimmu.2026.1777567)

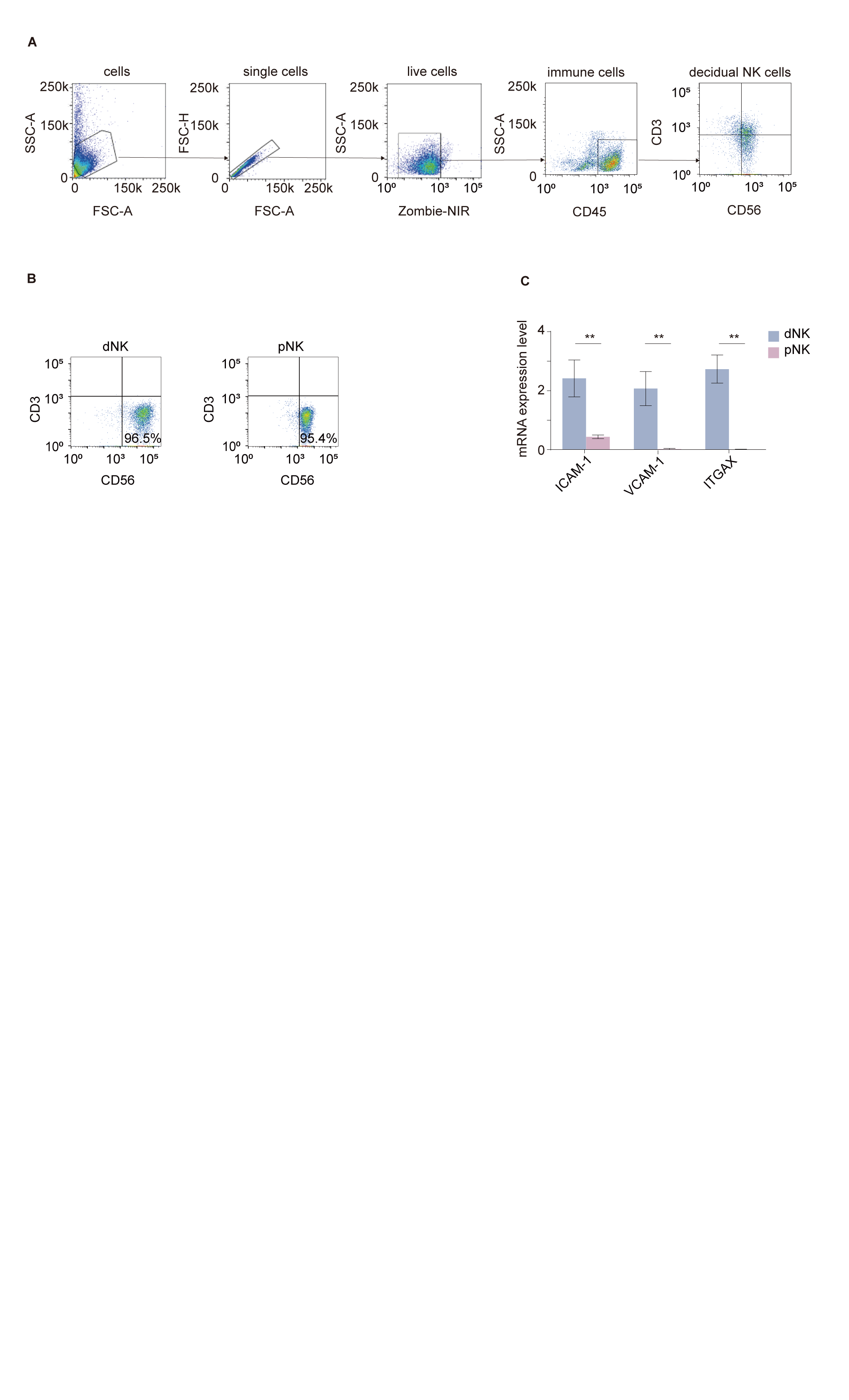

Supplement: Supplementary Figure 1 — Isolation and characterization of dNK and pNK cells and analysis of adhesion molecule expression. (A) Flow cytometry gating strategy within dNK. (B) The purity of isolated dNK and pNK cells assessed by flow cytometry (n = 6 per group). (C) Adhesion molecule (ICAM-1, VCAM-1 and ITGAX) mRNA expression in dNK and pNK cells (n = 10 per group). Data: mean ± SD; *P < 0.05, **P < 0.01, ns, not significant. [file Image1.tif]

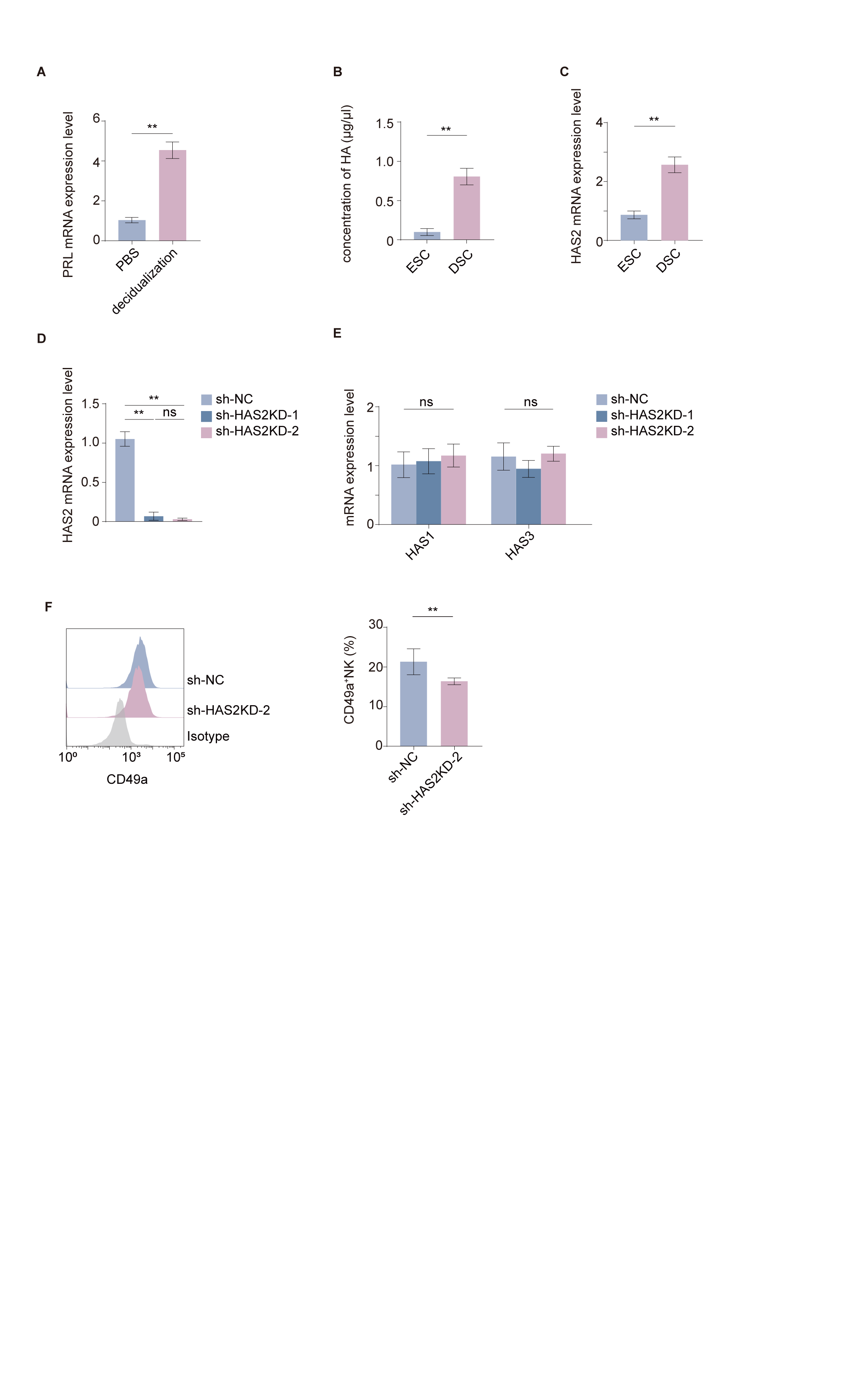

Supplement: Supplementary Figure 2 — Validation of HAS2 knockdown in DSCs and its effect on NK cell CD49a expression. (A) Prolactin expression in ESCs after decidualization treatment by RT-qPCR (n=6 per group). (B) HA concentration in DSC supernatant. (C) HAS2 expression in ESCs and DSCs (n = 6 per group). (D) Efficacy of two plasmids for the construction of DSCs with HAS2KD (n = 6 per group). (E) Expression of HAS1 and HAS3 in DSCs with HAS2KD (n = 6 per group) (F) CD49a expression in NK cells co−cultured with control DSCs or HAS2KD−2 DSCs (n = 6 per group). Data: mean ± SD; *P < 0.05, **P < 0.01, ns, not significant. [file Image2.tif]

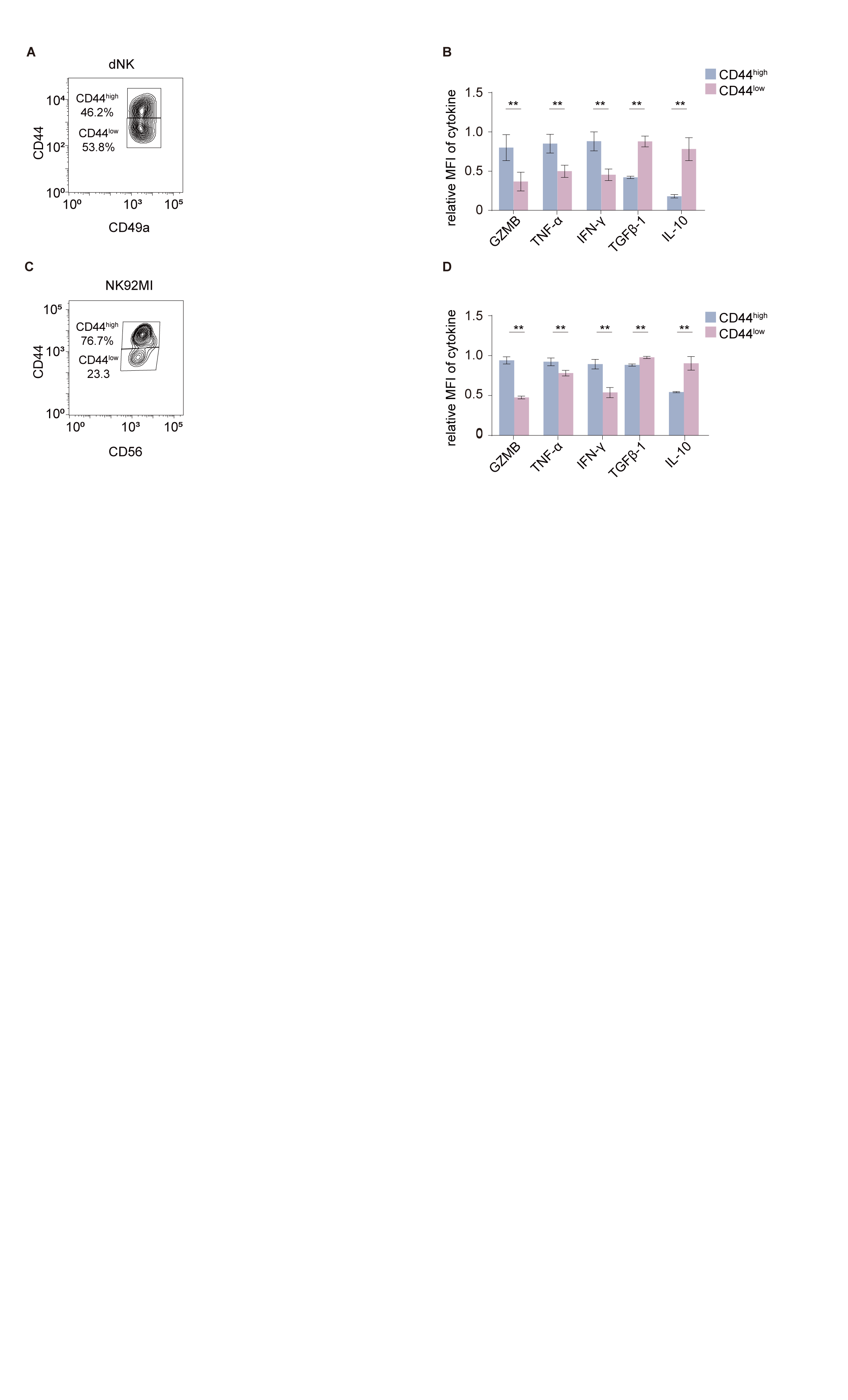

Supplement: Supplementary Figure 3 — CD44high and CD44low subpopulation definition and effector molecule profiles in dNK and NK92MI cells. (A) Division of dNK cells into CD44high and CD44low subpopulation. (B) Effect molecules (GZMB, TNF-α, IFN-γ, TGF-β1, IL-10) expression in CD44high and CD44low dNK cells by flow cytometry (n = 8 per group). (C) Division of NK92MI cells into CD44high and CD44low subpopulation. (D) Effect molecules (GZMB, TNF-α, IFN-γ, TGF-β1, IL-10) expression in CD44high and CD44low NK92MI cells by flow cytometry (n = 6 per group). Data are expressed as mean ± SD; *P < 0.05; **P < 0.01; ns, not significant. [file Image3.tif]
