# Supplementary material for: Hyaluronic acid−CD44 signaling from decidual stromal cells orchestrates dNK1 differentiation and immune tolerance in early pregnancy
Source: Front Immunol. 2026 Mar 25;17:1777567. doi: 10.3389/fimmu.2026.1777567 (PMC13057500; doi:10.3389/fimmu.2026.1777567)
Supplement: Supplementary file 4 [file Table1.docx]

Supplementary Table1. Polypeptide Sequence List

| Name | Sequences |
| --- | --- |
| HA inhibitory peptide | GAHWQFNALTVR |
| HA control peptide | WRHGFALTAVNQ |

Supplementary Table2. Flow Cytometry Reagent List

| Name | Channel | Brand | Item Number |
| --- | --- | --- | --- |
| anti-human CD56  anti-human CD49a  anti-human CD44  anti-human CD39  anti-human ITGB2  anti-human CD103  anti-human GZMB  anti-human GZMB  anti-human TNF-α  anti-human TNF-α  anti-human IFN-γ  anti-human TGF-β1  anti-human IL-10  anti-human IL-10 | FITC  PerCP/Cyanine5.5  BV510  PE  APC  BV421  PE  FITC  BV510  PE  APC  PE  APC  BV421 | Biolegend  Biolegend  Biolegend  BD  BD  BD  Biolegend  Biolegend  Biolegend  Biolegend  Biolegend  Biolegend  Biolegend  Biolegend | 318303  328322  103044  567157  551060  566257  372207  372205  502949  502908  502511  141404  506806  501421 |
| anti-human Ki67  Zombie-NIR  Human Trustain FcX  Mouse IgG1kIsotype Ctrl Antibody  Mouse IgG1kIsotype Ctrl Antibody  Mouse IgG1 kIsotype Ctrl Antibody  Mouse IgG1 kIsotype Ctrl Antibody  Mouse IgG1 kIsotype Ctrl Antibody  Mouse IgG1 kIsotype Ctrl Antibody  Rat IgG2a kIsotype Ctrl Antibody  Rat IgG2a kIsotype Ctrl Antibody | PE  APC/Cy7  -  FITC  PerCP/Cyanine5.5  BV510  PE  APC  BV421  APC  BV510 | Biolegend  Biolegend  Biolegend  Biolegend  Biolegend  Biolegend  Biolegend  Biolegend  BD  Biolegend  Biolegend | 350503  423106  422302  400137  400149  400171  141404  400141  569394  400511  400645 |

Supplementary Table3. Primer Sequences List

| Name | | Sequences(5’→3‘) |
| --- | --- | --- |
| ICAM-1 | Front: ATGCCCAGACATCTGTGTCC | |
|  | Reverse: GGGGTCTCTATGCCCAACAA | |
| VCAM-1 | Front: CAGTAAGGCAGGCTGTAAAAGA | |
|  | Reverse: TGGAGCTGGTAGACCCTCG | |
| ITGAX | Front: AGAGCTGTGATAAGCCAGTTCC | |
|  | Reverse: AATTCCTCGAAAGTGAAGTGTGT | |
| HAS1 | Front: CTGCGATGAGACAGGACGC | |
|  | Reverse: CTGAAAGGAAGGCCCCGTAG | |
| HAS2 | Front: CTCTTTTGGACTGTATGGTGCC | |
|  | Reverse: AGGGTAGGTTAGCCTTTTCACA | |
| HAS3 | Front: CAGAAGTCGGGGAAGAGTGC | |
|  | Reverse: CAGAGTCGCACACCTGGATG | |
| GZMB | Front: CCCTGGGAAAACACTCACACA | |
|  | Reverse: GCACAACTCAATGGTACTGTCG | |
| TNF-α | Front: CCTCTCTCTAATCAGCCCTCTG | |
|  | Reverse: GAGGACCTGGGAGTAGATGAG | |
| IFN-γ | Front: TCGGTAACTGACTTGAATGTCCA | |
|  | Reverse: TCGCTTCCCTGTTTTAGCTGC | |
| TGF-β1 | Front: GGCCAGATCCTGTCCAAGC | |
|  | Reverse: GTGGGTTTCCACCATTAGCAC | |
| Il-10  *STAT3*  *MAFB*  *HES1*  *FOSL2*  *ETV5*  GAPDH  tublin | Front: GACTTTAAGGGTTACCTGGGTTG | |
|  | Reverse: TCACATGCGCCTTGATGTCTG  Front: CAGCAGCTTGACACACGGTA  Reverse: AAACACCAAAGTGGCATGTGA  Front: TCAAGTTCGACGTGAAGAAGG  Reverse: GTTCATCTGCTGGTAGTTGCT  Front: TCAACACGACACCGGATAAAC  Reverse: GCCGCGAGCTATCTTTCTTCA  Front: AACACCCTGTTTCCTCTCCG  Reverse: ACCCGGAATTTCTGCTGGC  Front: TCAGCAAGTCCCTTTTATGGTC  Reverse: GCTCTTCAGAATCGTGAGCCA | |
|  | Front: GGAGCGAGATCCCTCCAAAAT | |
|  | Reverse: GGCTGTTGTCATACTTCTCATGG  Front: TTGGGAGGTCATCAGCGATGAG  Reverse: CTCCTTGCCAATGGTGTAGTGC | |
